# Differentiation of Post-Polio Syndrome from Prior Poliomyelitis Sequela by Assessing Paraspinal Muscle Involvement in Magnetic Resonance Imaging

**DOI:** 10.3390/jcm13164828

**Published:** 2024-08-16

**Authors:** Mahir Topaloglu, Deniz Sarikaya, Ahmet Peker, Yunus Emre Senturk, Rana Terlemez, Burak Ugur Cetin, Ali Emre Oge, Aysegul Ketenci

**Affiliations:** 1 Department of Physical Medicine and Rehabilitation, Koç University School of Medicine, 34450 Istanbul, Turkey; dsarikaya16@ku.edu.tr (D.S.); draysegulketenci@hotmail.com (A.K.); 2 Department of Radiology, Koç University School of Medicine, 34450 Istanbul, Turkey; apeker@kuh.ku.edu.tr (A.P.); ysenturk@kuh.ku.edu.tr (Y.E.S.); 3 Department of Physical Medicine and Rehabilitation, Cerrahpaşa Faculty of Medicine, Istanbul University, 34098 Istanbul, Turkey; ranakaynar@hotmail.com (R.T.); burakcetin000@hotmail.com (B.U.C.); 4 Department of Neurology, Koç University School of Medicine, 34450 Istanbul, Turkey; aemreoge@istanbul.edu.tr

**Keywords:** poliomyelitis, post-polio syndrome, paraspinal muscles, Mercuri scale, muscle magnetic resonance imaging

## Abstract

**Background/Objectives:** Post-polio syndrome (PPS) affects former polio patients, manifesting decades after initial infection with progressive symptoms like pain, fatigue, and muscle weakness. Diagnosis relies on the clinical criteria and exclusion of other probable causes. The purpose of this study is to determine the scope and new diagnostic value of magnetic resonance imaging (MRI) in identifying muscle involvement in PPS and distinguishing it from prior poliomyelitis (PPM). **Methods:** This study was approved by the Koç University Ethics Committee with Approval No. 2023.409.IRB2.090. Electronic medical archives from two academic institutions were searched for records tagged with ICD code B-91 for poliomyelitis sequalae. The resulting search query of 291 records was manually sorted for PPS and PPM, medical history, clinical examination findings, and lumbar MR images down to 32 patients. Two independent radiologists evaluated the paraspinal musculature in the MRIs using the Mercuri scale. Inter-rater agreement, comparison of the paraspinal musculatures between groups, and their relationship to leg involvement were assessed with the resulting data. **Results:** Inter-rater agreement was found to be almost perfect across all muscles, except for the multifidus muscle. When clinical examination findings were included for these muscles, quadratus lumborum (QL) degradation was found in both right-side (*p* = 0.017) and left-side (*p* = 0.002) leg involvement. **Conclusions:** QL muscle deterioration may serve as a diagnostic marker for PPS, potentially guiding lumbar pain treatment through rehabilitation.

## 1. Introduction

Post-polio syndrome (PPS) is a neuromuscular condition which affects individuals who were previously infected by the polio virus, the causative pathogen of poliomyelitis. Thanks to widespread immunization efforts led by the Global Polio Eradication Initiative, poliomyelitis was mostly eradicated by 1988 in developed countries [1,2]. However, many prior poliomyelitis (PPM) patients still carry the burden of the disease and may have to actively combat disease progression in the form of PPS.

This syndrome can occur up to 40 years after the initial polio infection and is often characterized by pain, additional fatigue, and progressive muscle weakness in both previously affected and unaffected regions [3]. The pathophysiological processes behind the syndrome are not well understood [4,5]. The diagnosis of PPS can be performed clinically along with the exclusion of other neurologic causes with the help of electroneuromyography (ENMG) and appropriate imaging studies. The clinical diagnosis of PPS is principally based on the March of Dimes criteria [6], which encompasses one’s previous infection history, stable status for more than 15 years, gradual and new onset of pain or fatigue which lasts more than 1 year, and exclusion of other conditions. Since the syndrome presents in a variety of clinical manifestations, the differential diagnoses also vary based on the presenting constellation of symptoms. In cases where progressive muscle weakness is involved, the clinician may have to consider disc herniations or compression neuropathies, whereas in cases where fatigue is more dominant, the clinician must also consider even the most basic culprits, such as hypothyroidism and anemia. It is argued that the current diagnostic criteria may not be sufficiently objective [3].

Patients with poliomyelitis and those who later develop PPS often experience significant impacts on their quality of life. Poliomyelitis survivors face long-term physical disabilities, including muscle weakness, fatigue, and mobility issues. These impairments persist for decades, affecting daily activities, career choices, and one’s overall well-being. For PPS patients, there is a secondary reduction in quality of life which typically occurs many years after the acute polio episode and can worsen one’s well-being. Severe reductions may lead to a further impact on mobility and activities of daily living, rendering self-care, work, and social participation more difficult. Additionally, PPS patients may face challenges in healthcare due to the complex nature of their condition and the need for multidisciplinary management approaches.

Compared with the extremity muscles typically assessed via physical examination and needle electromyography, the paraspinal muscles may be disregarded, despite their potential to provide critical clues in neuromuscular disorders. Challenges in evaluating paraspinal muscles contribute to this underuse, but previous studies investigating the role of magnetic resonance imaging (MRI) in various neuromuscular diseases and PPS have reported striking findings [7,8]. Diamanti et al. were able to differentiate between spinal onset and bulbar onset amyotrophic lateral sclerosis (ALS) by evaluating fat infiltration and atrophy in paraspinal musculature [9]. Klickovic et al. demonstrated that MRI can differentiate between ALS and spinal bulbar muscular atrophy [10]. The above-mentioned studies utilized the Mercuri scale to grade the extent of fatty infiltration into muscle tissue [11].

Within the broader scope of PPS, a previous study investigating PPS demonstrated a significantly higher difference in signal intensity under ultrasonographic evaluation when compared with healthy volunteers [12]. Gray matter atrophy has also been observed in the spinal cords of PPS patients when compared with healthy volunteers [13]. Another case report identified segmental denervation under MRI in a new-onset PPS patient [8].

The primary objective of the present study is to determine the scope of MRI in identifying muscle involvement associated with PPS. The secondary objective is to determine the diagnostic role of MRI in distinguishing PPS from PPM.

## 2. Methods

### 2.1. Participants and Data Collection

Ethics board approval for the retrospective use of institutional databases for this study was obtained from the Koç University Ethics Committee with Approval No. 2023.409.IRB2.090 on 14 December 2023. For this retrospective analysis, the ethics board determined that no informed consent was necessary.

We conducted this cross-sectional study on the electronic medical record archives of Koc University Hospital and Istanbul University-Cerrahpasa Faculty of Medicine for patients tagged with ICD-10 code B-91 (Poliomyelitis Sequalae) between 1 January 2015 and 1 April 2024. The resulting records were manually sorted for demographic variables as well as the history of PPM or PPS, medical history with comorbidities, affected limbs, mobilization, muscle strength, use of assistive devices (orthotics, walkers, etc.), ENMG reports, and MRI files. Patients who were diagnosed with poliomyelitis based on electrophysiologically demonstrated lower motor neuron involvement who were between the ages of 18 and 65 years and able to walk alone with or without an assistive device were included in this study. Immobile patients and those with peripheral muscle strength of less than 3/5 were excluded to avoid bias from associated atrophy. Patients younger than 18 years were excluded, as these patients would have been born after the eradication of poliomyelitis. Absent or technically inaccessible MRI files were omitted from this study. Patients with matching medical histories as well as records that fulfilled the March of Dimes criteria [6] were categorized into the PPS subgroup.

Radiologic images for the age- and gender-adjusted control groups for mechanical lower back pain (LBP) were supplied by the Department of Radiology of Koc University Hospital using previous images with ICD-10 codes M54.1 for radiculopathy and M54.5 for mechanical LBP without radiculopathy. The medical records of these patients were evaluated, and those with L2 or L3 radiculopathies were excluded.

### 2.2. MRI Data Analysis

As raters, two independent radiologists with 10 and 11 years of radiological expertise were assigned. Rater 1 was a musculoskeletal radiologist (A.P.) with 5 years of particular expertise in spinal radiology, and Rater 2 was a neuroradiologist (Y.E.S.) with 6 years of experience in the field of neuromuscular disorders. They evaluated each image blinded to the clinical data independently and assessed paraspinal musculatures based on the Mercuri scale [10] (Table 1). To prevent recall and potential evaluation bias, all muscle scorings were performed in a random sequence during a single session by each radiologist.

**Figure 1:**
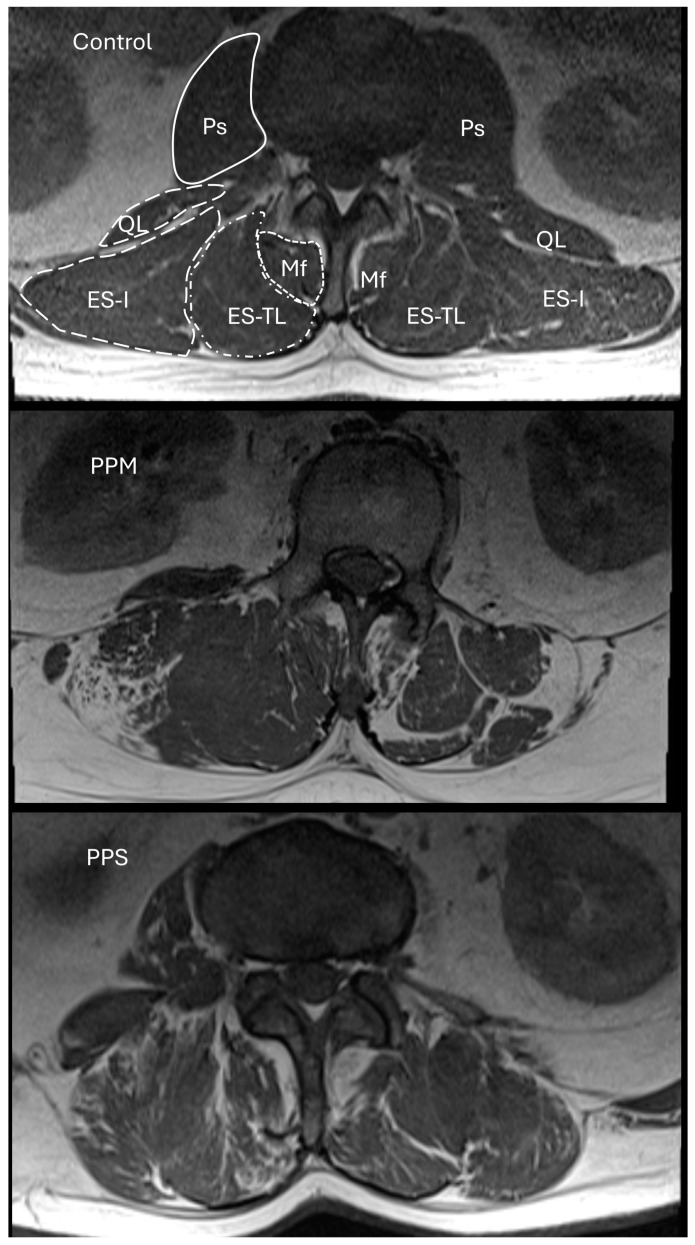
Axial images of lumbar paraspinal musculature in control, prior poliomyelitis (PPM), and post-polio syndrome (PPS) patients. Ps = psoas, QL = quadratus lumborum, Mf = multifidus, ES-I = erector spinae iliocostalis, ES-TL = erector spinae thoracicus longus.

The Mercuri scale categorizes MRI findings into 5 groups with scores ranging from 0 to 4, with two subgroups for 2a and 2b. The evaluation regions were determined to be the psoas (Ps), quadratus lumborum (QL), multifidus (Mf), erector spinae pars iliocostalis (ES-I), and erector spinae pars thoracicus longus (ES-TL) muscles bilaterally at the L2 vertebra level. The evaluated sections for the L2–L3 intervertebral disc level are depicted in Figure 1.

Since patient records were recruited from multiple centers, the MRI sequences were not identical. Some of the images were obtained with 3T or 1.5T scanners. The patients’ MR scans included axial T2-weighted TSE sequences with a slice thickness varying between 3 and 4 mm, as well as sagittal T2- and T1-weighted TSE sequences with a thickness varying between 3 and 5 mm. The determination of the L2–L3 intervertebral disc level was performed using the sagittal images. The paraspinal musculature was evaluated using axial T2-weighted TSE sequences.

### 2.3. Statistical Analysis

Descriptive statistics were used to describe the study population. Inter-rater agreement was evaluated using the weighted kappa statistic due to the ordinal nature of the Mercuri scale. The Kruskal–Wallis H test was employed to evaluate differences in the muscle measurements. Spearman’s rank correlation was used to assess the correlation between muscle involvement severity in the MRI (according to the Mercuri scale) and the presence of clinical leg involvement as PPM sequela or as weakness, atrophy, or other signs resulting from PPS. Due to the retrospective nature of this study, we did not aim to search for correlations based on the degree of muscle weakness or atrophy; merely the presence and side of clinical leg involvement were used for searching for correlations between the clinical and MRI involvements in the statistical analyses. All statistical analyses were conducted using Python 3 (Python Software Foundation, Wilmington, DE, USA, 2009) with the NumPy, Pandas, and SciPy statistics libraries [14,15,16], and visualizations were created using the Matplotlib library [17].

## 3. Results

**Figure 2:**
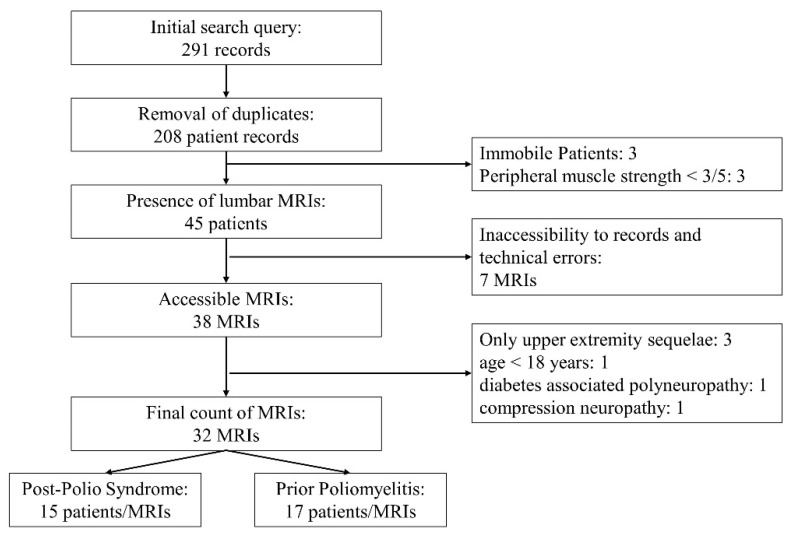
Flow chart of study design.

The electronic medical record archives from the two institutions revealed a total of 291 individual examination records with ICD tags indicating a history of poliomyelitis. Consolidating multiple examination records from the same query yielded a total number of 208 individual patients. From these, 45 patients had records mentioning a previous lumbar MRI. Using the national health registry’s database as well as local institutional databases, a total of 38 PPM or PPS patients with MRI images of the lumbar spine were identified. Three patients were excluded due to clinical involvement in only the upper extremities, one was excluded due to their age being below 18 years, and two were excluded due to ENMG findings indicating additional diabetes associated polyneuropathy and compression neuropathy. A flow chart summary of the recruitment is depicted in Figure 2.

A total of 32 unique lumbar MRIs from PPM and PPS patients were identified, these are summarized in Table 2. The PPS group comprised 15 participants (8 female and 7 male) with a median imaging age of 51 years. The PPM group included 17 participants (5 female and 12 male) with a median imaging age of 52 years. The PPS group exhibited 11 right leg sequalae, 8 left leg sequalae, and 4 bilateral leg sequalae, while the PPM group demonstrated 12 right leg sequalae, 8 left leg sequalae, and 3 bilateral leg sequalae, indicating a higher prevalence of right leg sequalae in both groups.

The inter-rater agreement findings for the study and control groups are depicted in Table 3. Inter-rater agreement was found to be almost perfect (>0.810) for all muscle ratings, except for the right multifidus in the polio groups, where substantial agreement was found (0.610–0.810). Inter-rater agreement in the LBP (stratified for both radiculopathy and lower back pain) group was almost perfect (>0.810) across all muscles. As a consequence, the ratings of one of the radiologists (Rater 1) were used in the analyses.

Intra-group analysis of the LBP patients revealed no significant difference between the radiculopathy and non-radiculopathy patients. The muscle ratings between the PPS and control groups showed significant differences for all muscles, but comparison of the PPS and PPM patients resulted in no observable difference in any parameter (Table 4).

**Figure 3:**
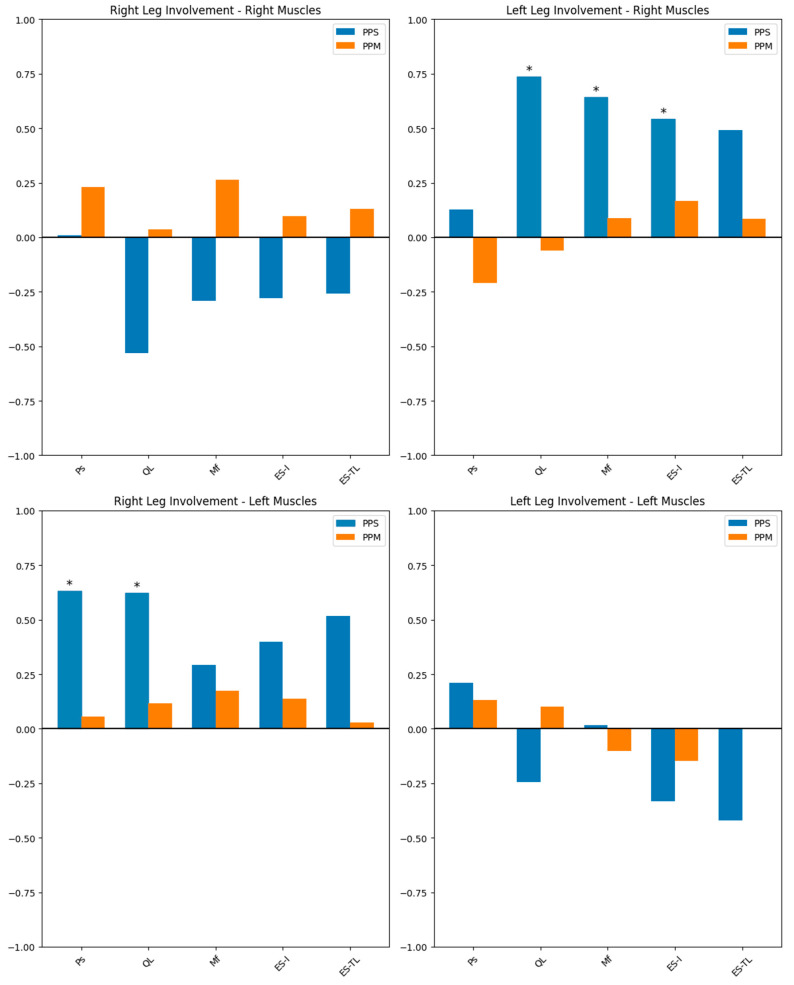
Correlation of leg involvement with ipsilateral and contralateral muscle ratings using Spearman rank correlation tests. Upward bars indicate positive correlations, and downward bars show negative correlations. Asterisks (*) denote statistical significance (*p* < 0.05). Ps = psoas, QL = quadratus lumborum, Mf = multifidus, ES-I = erector spinae iliocostalis, ES-TL = erector spinae thoracicus longus.

Correlations between the presence of leg involvement and the severity of MRI muscle involvement are displayed on Table 5. Figure 3 contains four plotted graphs for the side of clinical leg involvement and MRI analyses of ipsilateral and contralateral musculature. For both Table 5 and Figure 3, negative values represent an inverse relationship, and increasing values imply higher Mercuri scores and therefore more advanced muscle involvement in the MRI. The PPM patients showed no significant correlation across all muscle groups and clinical involvement.

In the setting of PPS, QL involvement was found to be statistically correlated with both right-side (*p* = 0.017) and left-side (*p* = 0.002) leg involvements contralaterally. The PPS patients with right leg involvement also showed statistically significant MRI involvement of contralateral Ps (*p* = 0.015), whereas left leg involvement showed the additional MRI involvement of contralateral ES-I (*p* = 0.036) and Mf (*p* = 0.01). The PPS patients with right leg sequelae also showed a nearly significant rating of ipsilateral QL (*p* = 0.05) in favor of inverse correlation, and a similar yet non-significant inverse correlation was also found in the left leg sequelae.

## 4. Discussion

In the current study, we aimed to evaluate paraspinal muscle involvement with MRI in patients with prior PPM, including those having PPS. Our findings primarily highlight QL MRI deterioration contralateral to the involved legs in PPS patients, suggesting that this phenomenon may be a predictive sign in the diagnosis of PPS.

Our study found a high level of inter-rater agreement when comparing muscle ratings in the PPS + PPM and control groups, as depicted in Table 3. Previous studies utilized Cohen’s kappa to assess the inter-rater agreement between Mercuri scores. We chose to utilize the weighted kappa as it better suits the ordinal nature of Mercuri ratings in addition to highlighting the extent of agreements or disagreements between raters. Post hoc analysis of our data revealed no decrease in agreement when using Cohen’s kappa instead. Minor variations in ES-TL and ES-I on both sides across both raters may be attributable to the smaller muscle body cross section on the imaging as well as their more superior insertion. Therefore, there was redundant innervation from superior nerve roots in terms of compensating poliomyelitis progression from inferior roots [18].

Lumbar paraspinal muscles consist of several muscle groups with different innervation patterns. The multifidus muscle, located more medially, is innervated by a single nerve root at each level, while the longissimus dorsi and iliocostalis muscles are innervated by multiple nerve roots. The deeper quadratus lumborum and psoas muscles are also crucial stabilizers of the vertebral column. However, their anatomical position makes them challenging when performing ENMG [19]. The evaluation of these muscles may be indicative in diagnosing pathological conditions, as fat infiltration in the paraspinal muscles varies among individuals due to different factors. For instance, it is widely accepted that paraspinal muscle degeneration is correlated with degenerative disc diseases [20].

A combined evaluation of clinical leg involvement with MRI muscle rating revealed significant involvement of the contralateral QL musculature. This makes identifying significant changes in tissue imaging of QL potentially important, since this muscle is relatively less affected by vertebral pathologies and may therefore be a candidate marker of PPS. Inter-rater reliability of QL imaging was found to be excellent (1.000) in the control groups as well as near excellent (0.981) in the PPM and PPS groups. On the other hand, our findings support one of the theories on PPS pathophysiology which blames the breakdown of compensatory adaptations or stabilizations which occurs in some of the less involved muscles, including QL, following the initial onset [5]. In this sense, muscle weakness in PPS progresses slowly as motor units progressively drop off in muscles which are critical in sustaining stability and mobilization [5]. Non-ubiquitous findings regarding contralateral Mf, Ps, and ES-I involvement highlight the potentially complex nature of PPS-associated muscle involvement and compensatory mechanisms.

Paraspinal MRI has become increasingly important in diagnosing several neuromuscular pathologies, particularly those causing selective fatty replacement in these muscles. Unlike the non-selective fatty changes seen in degenerative disc diseases, selective fatty replacement can be indicative of specific conditions. For instance, Diamanti et al. reported that selective fatty replacement of the longissimus dorsi muscle can play a diagnostic role in patients with motor neuron diseases [9].

When evaluating paraspinal muscles with imaging alone, both raters were unable to differentiate PPS from PPM, as no significant difference was observed (Table 4). On the other hand, a comparison between PPM or PPS and the control group showed significant differences regardless of the side of leg involvement, as expected from the nature of the disorder [7,10,20].

Interestingly, unaffected limb sides in PPM showed a slight degree of MRI involvement as well (Table 5 and Figure 3). Logically, we expected these sides to be less affected due to compensatory hypertrophy. Furthermore, a statistically non-significant compensatory muscle improvement can be interpreted from ipsilateral QL inverse correlation with leg sequelae (*p* = 0.5 and 0.379). Unlike QL, erector spinae muscles typically show atrophy in patients with radiculopathy and would therefore complicate identification [21].

Given the decreased prevalence of PPM, the major strengths of this study are the size of the PPM and PPS groups and the in-depth access to medical records. Regarding weaknesses, the retrospective nature of this study as well as a potential bias arising from documentation may be critiques of inferiority in contrast to a prospective case series study. Furthermore, utilization of images from various MRI machinery is also a drawback and prohibits standardized and objective analysis of the muscle content and signal intensity. Although all patients within the LBP group with radiculopathy had intervertebral disc herniations on either the L4–L5 or L5-S1 levels, we were unable to stratify the RP group based on the herniation level nor the localization or ENMG validation.

The utilization of platform-based rehabilitation interventions, such as whole-body vibration exercises, appears to present a rational approach for targeting QL muscle rehabilitation. This approach was investigated in a randomized controlled trial and was found to show efficacy in mitigating fatigue without inducing muscular damage [22]. This finding suggests that platform-based exercises may offer a viable, non-damaging modality for QL muscle rehabilitation. Further research could elucidate optimal application parameters. 

## 5. Conclusions

Patients with both prior poliomyelitis and post-polio syndrome showed significant MRI changes in their paraspinal musculature in contrast to the control groups with LBP. Evaluation of one’s paraspinal musculature using the Mercuri scale proved to be reliable and reproducible between two raters in the setting of PPS and PPM sequelae.

Our findings suggest that QL deterioration in PPS may serve as a diagnostic marker for the syndrome. This deterioration was significant when correlated with the presence of leg sequelae, highlighting muscle involvement in the contralateral QL muscles in PPS patients. This discovery raises the question of whether lumbar pain associated with PPS can be treated by addressing the involvement of QL by means of rehabilitation.

The discovery of significant deterioration in the QL musculature of PPS patients has important implications for rehabilitation strategies and overall patient care. As QL plays a major role in spinal stabilization, respiration, and lateral flexion of the trunk, we identified a focus of rehabilitation to prophylactically target and alleviate the impairments commonly observed in PPS.

From a rehabilitation perspective, this discovery would necessitate a reevaluation of current treatment approaches. Physiatrists may need to develop or avoid specific exercises and techniques to address quadratus lumborum weakness. These might include targeted strengthening exercises, core stability training, and postural re-education. However, it would be crucial to balance the need for muscle strengthening with the risk of overuse, as excessive exercise can lead to further deterioration in PPS patients.

These findings could also influence the design of assistive devices and orthotics. Braces or supports which provide additional lumbar stabilization might be beneficial in compensating for quadratus lumborum weakness. Moreover, this knowledge could inform ergonomic recommendations for PPS patients, potentially leading to modifications in seating, work environments, and daily activity patterns to reduce strain on the affected muscles.

From a medical management standpoint, healthcare providers might need to pay closer attention to the quadratus lumborum during patient assessments. Regular monitoring of this muscle’s function could become an important aspect of PPS follow-up care. Additionally, this discovery might spark new avenues for research into potential pharmacological or regenerative therapies targeting muscle preservation or regeneration in PPS patients.

Lastly, these findings underscore the importance of a multidisciplinary approach to PPS management. Collaboration between neurologists, physiatrists, physiotherapists, and occupational therapists would be crucial in developing comprehensive treatment plans which address quadratus lumborum deterioration while considering the overall impact on a patient’s functional capacity and quality of life.

Further prospective studies as well as analyses involving the degree and extent of clinical involvement and MRI examinations at different vertebral levels are required to investigate changes in MRI findings on PPM sequelae patients during the potential onset of PPS. This approach would help to evaluate the sensitivity and specificity of assessing the worsening QL musculature and its role in lumbar pain management in PPS patients.

## Figures and Tables

**Table 1:** Description of the qualitative muscle grading scale as defined by Mercuri et al. [11].

Grade	Mercuri Scale
0	Normal appearance
1	Early moth-eaten appearance with scattered small areas of increased signal
2a	Late moth-eaten appearance with numerous discrete areas of increased signal with beginning confluence, comprising less than 30% of the volume of the individual muscle
2b	Late moth-eaten appearance with numerous discrete areas of increased signal with beginning confluence, comprising 30–60% of the volume of the individual muscle
3	Washed-out, fuzzy appearance due to confluent areas of increased signal
4	End stage appearance, with muscle replaced by increased density of connective tissue and fat and only a rim of fascia and neurovascular tissue distinguishable

**Table 2:** Summary of demographic findings between the polio and post-polio groups. IQR = interquartile range.

Group	Participants	Female	Male	Median Imaging Age (Years)	IQR (Years)	Right Leg Sequelae	Left Leg Sequelae	Bilateral Leg Sequelae
PPS	15	8	7	51	5	11	8	4
PPM	17	5	12	52	18	12	8	3

**Table 3:** Inter-rater agreement based on weighted kappa statistic for the study groups. PPS = post-polio syndrome, PPM = prior poliomyelitis, R = right, L = left, Ps = psoas, QL = quadratus lumborum, Mf = multifidus, ES-I = erector spinae iliocostalis, ES-TL = erector spinae thoracicus longus.

Inter-Rater Agreement		
Muscle	PPS and PPM Groups	Control Group
R Ps	0.982	1.000
L Ps	0.978	1.000
R QL	0.920	1.000
L QL	0.981	1.000
R Mf	0.719	0.919
L Mf	0.865	0.919
R ES-I	0.903	0.826
L ES-I	0.824	0.826
R ES-TL	0.832	0.838
L ES-TL	0.833	0.838

**Table 4:** Comparison of muscle evaluation for post-polio versus prior poliomyelitis and lower back pain control groups using Kruskal–Wallis test. Asterisks (*) denote statistical significance. Ps = psoas, QL = quadratus lumborum, Mf = multifidus, ES-I = erector spinae iliocostalis, ES-TL = erector spinae thoracicus longus.

	PPS vs. PPM		PPS vs. Control	
Muscle	Rater 1 Statistic	Rater 1 *p* Value	Rater 1 Statistic	Rater 1 *p* Value
R Ps	1.0437	0.3070	57.718	* < 0.01
L Ps	0.6050	0.4367	63.245	* < 0.01
R QL	0.9372	0.3330	28.921	* < 0.01
L QL	0.1294	0.7191	50.708	* < 0.01
R Mf	2.2031	0.1377	14.687	* < 0.01
L Mf	1.7899	0.1809	23.34	* < 0.01
R ES-I	0.2133	0.6442	6.553	*0.0105
L ES-I	0.2196	0.6394	9.264	* < 0.01
R ES-TL	0.9036	0.3418	6.686	* < 0.01
L ES-TL	0.5351	0.4645	8.19	* < 0.01

**Table 5:** Spearman’s rank correlation of muscle sequelae with leg involvement laterality for post-polio groups and poliomyelitis groups. PPS = post-polio syndrome, PPM = poliomyelitis, R = right, L = left, Ps = psoas, QL = quadratus lumborum, Mf = multifidus, ES-I = erector spinae iliocostalis, ES-TL = erector spinae thoracicus longus. Asterisks (*) denote statistical significance (*p* < 0.05).

Muscle Group	Leg Involvement	PPS Correlation	*p* Value	PPM Correlation	*p* Value
R Ps	Right	0	1	0.231	0.373
	Left	0.126	0.655	−0.209	0.455
L Ps	Right	0.634	* 0.015	0.056	0.83
	Left	0.209	0.454	0.131	0.641
R QL	Right	−0.532	0.05	0.035	0.893
	Left	0.737	* 0.002	−0.06	0.833
L QL	Right	0.623	* 0.017	0.115	0.66
	Left	−0.245	0.379	0.101	0.719
R Mf	Right	−0.291	0.313	0.263	0.309
	Left	0.643	* 0.01	0.087	0.757
L Mf	Right	0.291	0.313	0.173	0.507
	Left	0.016	0.955	−0.101	0.719
R ES-I	Right	−0.281	0.331	0.098	0.708
	Left	0.544	* 0.036	0.166	0.554
L ES-I	Right	0.397	0.159	0.139	0.595
	Left	−0.333	0.225	−0.149	0.597
R ES-TL	Right	−0.257	0.375	0.13	0.618
	Left	0.492	0.063	0.086	0.761
L ES-TL	Right	0.517	0.058	0.029	0.913
	Left	−0.419	0.12	0	1

## Data Availability

The original contributions presented in the study are included in the article, further inquiries can be directed to the corresponding author.
